# Validation of In Vivo Nodal Assessment of Solid Malignancies with USPIO-Enhanced MRI: A Workflow Protocol

**DOI:** 10.3390/mps5020024

**Published:** 2022-03-07

**Authors:** Daphne A. J. J. Driessen, Didi J. J. M. de Gouw, Rutger C. H. Stijns, Geke Litjens, Bas Israël, Bart W. J. Philips, John J. Hermans, Tim Dijkema, Bastiaan R. Klarenbeek, Rachel S. van der Post, Iris D. Nagtegaal, Adriana C. H. van Engen-van Grunsven, Lodewijk A. A. Brosens, Andor Veltien, Patrik Zámecnik, Tom W. J. Scheenen

**Affiliations:** 1 Department of Radiation Oncology, Radboud University Medical Center, 6525 Nijmegen, The Netherlands; tim.dijkema@radboudumc.nl; 2 Department of Surgery, Radboud University Medical Center, 6525 Nijmegen, The Netherlands; didi.degouw@radboudumc.nl (D.J.J.M.d.G.); rutger.stijns@radboudumc.nl (R.C.H.S.); bastiaan.klarenbeek@radboudumc.nl (B.R.K.); 3 Department of Medical Imaging, Radboud University Medical Center, 6525 Nijmegen, The Netherlands; g.litjens@radboudumc.nl (G.L.); bas.israel@radboudumc.nl (B.I.); bartphilips1@gmail.com (B.W.J.P.); john.hermans@radboudumc.nl (J.J.H.); andor.veltien@radboudumc.nl (A.V.); patrik.zamecnik@radboudumc.nl (P.Z.); tom.scheenen@radboudumc.nl (T.W.J.S.); 4 Department of Pathology, Radboud University Medical Center, 6525 Nijmegen, The Netherlands; chella.vanderpost@radboudumc.nl (R.S.v.d.P.); iris.nagtegaal@radboudumc.nl (I.D.N.); ilse.vanengen-vangrunsven@radboudumc.nl (A.C.H.v.E.-v.G.); lodewijk.brosens@radboudumc.nl (L.A.A.B.)

**Keywords:** magnetic resonance imaging, nodal staging, lymph node, 7 Tesla, node-to-node correlation, pathology

## Abstract

Background: In various cancer types, the first step towards extended metastatic disease is the presence of lymph node metastases. Imaging methods with sufficient diagnostic accuracy are required to personalize treatment. Lymph node metastases can be detected with ultrasmall superparamagnetic iron oxide (USPIO)-enhanced magnetic resonance imaging (MRI), but this method needs validation. Here, a workflow is presented, which is designed to compare MRI-visible lymph nodes on a node-to-node basis with histopathology. Methods: In patients with prostate, rectal, periampullary, esophageal, and head-and-neck cancer, in vivo USPIO-enhanced MRI was performed to detect lymph nodes suspicious of harboring metastases. After lymphadenectomy, but before histopathological assessment, a 7 Tesla preclinical ex vivo MRI of the surgical specimen was performed, and in vivo MR images were radiologically matched to ex vivo MR images. Lymph nodes were annotated on the ex vivo MRI for an MR-guided pathological examination of the specimens. Results: Matching lymph nodes of ex vivo MRI to pathology was feasible in all cancer types. The annotated ex vivo MR images enabled a comparison between USPIO-enhanced in vivo MRI and histopathology, which allowed for analyses on a nodal, or at least on a nodal station, basis. Conclusions: A workflow was developed to validate in vivo USPIO-enhanced MRI with histopathology. Guiding the pathologist towards lymph nodes in the resection specimens during histopathological work-up allowed for the analysis at a nodal basis, or at least nodal station basis, of in vivo suspicious lymph nodes with corresponding histopathology, providing direct information for validation of in vivo USPIO-enhanced, MRI-detected lymph nodes.

## 1. Introduction

Many different types of solid cancers have a high propensity to metastasize to locoregional and distant lymph nodes, which is one of the most important prognostic factors for survival [1,2]. Current clinical practice employs considerable overtreatment for precautionary reasons. If an accurate pre-treatment lymph node status is established, therapeutic strategies can be tailored to the individual patient. With continuously improving opportunities for selective treatment of individual metastatic deposits with surgery or radiotherapy, assessment and exact localization of lymph node metastases is crucial. Concurrently, increased certainty of the absence of lymph node metastases could safely minimize treatment. Therefore, highly sensitive detection of small lymph node metastases is urgently needed [3].

Compared to other imaging techniques, magnetic resonance imaging (MRI) provides superior soft-tissue contrast. This allows for good visualization of small anatomical structures, and it can be used to characterize lymph nodes [4]. MRI, in combination with a contrast agent of ultrasmall superparamagnetic particles of iron oxide (USPIO), might be a promising, generalized, diagnostic tool to detect lymph node metastases in various types of cancers [5,6,7,8,9]. When compared to other diagnostic imaging techniques, USPIO-enhanced MRI does not merely rely on the size and shape of the lymph node but provides a contrast between the benign and malignant part of a lymph node [10,11]. In short, 24–36 hours after intravenous administration, normal lymph nodes accumulate USPIO nanoparticles in macrophages, causing a strong attenuation of the MR signal, while suspicious lymph nodes without USPIO retain MR signal on T2* weighted sequences [12]. Promising results were previously reported in different body regions with a pooled sensitivity of 0.90 and a pooled specificity of 0.96 [13]. Nevertheless, the clinical value of USPIO-enhanced MRI using state-of-the-art 3-dimensional (3D) sequences with high isotropic spatial resolution should be appropriately validated [14,15]. This demands for accurate validation of USPIO-enhanced MRI compared to histopathology on a nodal basis.

Few attempts have been undertaken to match individual lymph nodes on MRI with the corresponding lymph node on histopathology after lymphadenectomy [16,17]. Al-though results were promising, correlation of lymph nodes on a node-to-node basis remains difficult, and small lymph nodes (<3mm) on in vivo MRI often cannot be correlated with pathology. A seven Tesla (T) ex vivo MRI can enable visualization of the detailed anatomy of the resected specimen with the exact locations of dissected lymph nodes. Here, we present a protocol we developed to correlate in vivo visible lymph nodes in various body regions with histopathology data on a nodal basis using 7T ex vivo MRI of lymphadenectomy specimens as an intermediate step. This is a way to validate in vivo MRI techniques with histopathology on a nodal basis to improve nodal staging.

## 2. Materials and Methods

### 2.1. Subjects

The data were collected from one trial investigating ex vivo MR scanning of the resection specimen in prostate cancer patients who underwent USPIO-enhanced MRI in the clinical setting and from four trials that investigate the feasibility of USPIO-enhanced MRI for detecting suspicious lymph nodes (rectal cancer NCT02751606 [18], pancreatic and periampullary cancer NCT04311047, esophageal cancer NTR6072, and head-and-neck cancer NCT03817307). To illustrate the feasibility of the proposed workflow, one patient for each of the five types of solid malignancies was presented as an example. Characteristics of all patients are described in Table 1. All studies were approved by the central ethics committee on research involving human subjects and the local ethics committee on research involving human subjects, Arnhem-Nijmegen. Signed informed consent was obtained before patient inclusion according to each corresponding research protocol. Patients were enrolled at the Radboud university medical center between September 2017 and June 2019 by the coordinating investigator or treating physician. An overview of the inclusion and exclusion criteria for each study protocol has been attached in a separate file.

In all patients, 2.6 mg/kg body weight of USPIO (Ferumoxtran-10, Ferrotran^®^ SPL Medical B.V. Nijmegen, the Netherlands) mixed with 0.9% saline solution was administered intravenously with a slow-drip infusion of 30–45 min under continuous clinical supervision. Then, 24–36 hours later, an MRI examination was performed using a 3T MR system (Magnetom Skyra or Prisma, Siemens Healthcare, Erlangen, Germany). Sequences were focused on detecting USPIO in lymph nodes and consisted of a 3D T1-weighted volumetric interpolated breath-hold examination (VIBE) Dixon pulse sequence and 3D multi gradient echo (MGRE) sequence [19]. The MRI pulse sequence parameters are described in Table 2 and Table 3. All patients, except for the head-and-neck cancer patient, received intramuscular smooth muscle and bowel relaxant (scopolamine butyl bromide, Sanofi-aventis Netherlands B.V.) to reduce peristaltic motion. The periampullary cancer patients also received glucagon to reduce peristaltic motion.

### 2.2. Surgery and Ex Vivo MR Examination

Resection of the primary tumor and lymphadenectomy was performed according to routine clinical procedures. Immediately after resection, the specimen was transported at room temperature from the operating room to the Department of Pathology. The surgical specimen was, according to each different study protocol, either fixated with formalin before (prostate, rectal, esophageal, and head-and-neck cancer) or after ex vivo MRI (periampullary cancer). This ex vivo MRI was performed on a 7T preclinical MR system using a volume radiofrequency coil (ClinScan, Bruker® BioSpin, Ettlingen, Germany). The specimen was placed in a proximal-distal position in the preclinical MR system. The MR protocol consisted of a T1-weighted 3-dimensional GRE sequence with frequency-selective lipid excitation and a 3D multi-GRE pulse sequence with five acquired echoes and frequency selective water excitation. The ex vivo MR sequence parameters are described in Table 4. All surgical specimens with a size exceeding 10 cm were scanned in multiple contiguous sections and were eventually merged to one composed MR image dataset. MeVisLab software (MeVis Medical Solutions, Fraunhofer MEVIS) was used to view and annotate the 3D ex vivo composed lipid and water-selective images simultaneously. All structures that were spherical and bordered in three dimensions were identified as lymph nodes by an experienced coordinating investigator.

The ex vivo MRI was used as an essential step for matching lymph nodes between in vivo MRI and histopathology, not for assessment of lymph node status on the basis of the presence or absence of USPIO. The interval between USPIO administration and surgery varied between 1 and 9 days (Table 1). In our experience, USPIO remained visible on MR-images until 2–3 days after infusion. The effect of iron particle accumulation in healthy (parts of) lymph nodes was only visible when the ex vivo MRI was performed within this timeframe. Visualizing USPIO accumulation in ex vivo MRI is not a prerequisite in the workflow we propose here, as the ex vivo MRI is used as an intermediate step to match any lymph node from in vivo MRI to the dissection specimen and thus to histopathology. Localizing a lymph node on in vivo MRI and matching it to the corresponding nodal structure on ex vivo MRI of the resected specimen was primarily executed on the basis of nodal size, nodal shape, and nodal location in relation to anatomical landmarks rather than MRI signal intensity changes.

### 2.3. MRI-Guided Pathology and Node-to-Node Correlation

In vivo MR images were evaluated by one or two radiologists and the researcher coordinating the study, annotating visible lymph nodes in the area of interest. After the ex vivo MRI of resected tissue, the in vivo annotated lymph nodes were cognitively matched by the experienced coordinating investigator of each study in conjunction with a radiologist to the ex vivo MRI data. On the basis of all 3D MRI scans with multiple contrasts, the lymph node locations were matched with respect to anatomical landmarks that were visible in the tissue on both in vivo and ex vivo scans. Individual nodal matching was performed on the basis of lymph node characteristics such as size, shape, location in relation to anatomical landmarks (such as a large vessel, its relation to the primary tumor, or the nodal level it was situated in), and nodal enhancement pattern in case the time between infusion and ex vivo MRI was <2–3 days. Subsequently, the lymphadenectomy specimen was dissected at the Department of Pathology with the 3D ex vivo MR images with annotated nodes presented on-screen to the pathologist [18,20]. Freedom to move through the 3D MRI datasets in all orientations allowed for the guidance of the pathologist to localize and dissect the annotated lymph nodes. The annotated lymph nodes were located in the specimen, for example, by measuring the distance between a lymph node and an edge of the resection specimen or an anatomical landmark. Subsequently, this lymph node was taken out and separately enclosed in a tissue cassette. The lymph nodes were numbered accordingly on in vivo MRI, ex vivo MRI, and the corresponding tissue cassette. All lymph nodes were, when larger than 5.0 mm, sectioned multiple times in 2.0–3.0 mm thick sections for paraffin embedding. All nodes were examined with a hematoxylin and eosin (H&E) stain on 4 μm sections. Relating the histopathological slide to the corresponding ex vivo-assigned lymph node and subsequently to the corresponding in vivo-identified lymph node provided the complete node-to-node correlation.

## 3. Results

**Figure 1:**
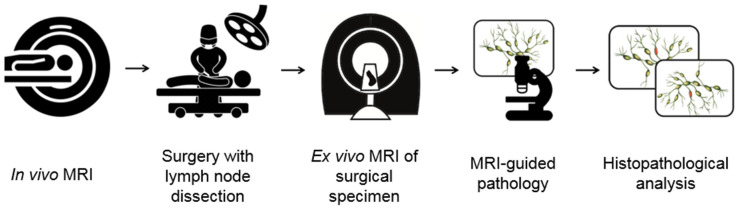
Schematic overview of the workflow.

In vivo USPIO-enhanced MRI and ex vivo MRI of resected specimens was technically feasible in all five patients. The general workflow to improve nodal staging for solid malignancies of different anatomical origins is schematically visualized in Figure 1. Due to different clinical routines in pathological work-up for each primary tumor localization, each tumor-specific workflow currently developed has differences in approach. These specific steps are outlined below for each primary tumor localization.

### 3.1. Prostate Cancer

**Figure 2:**
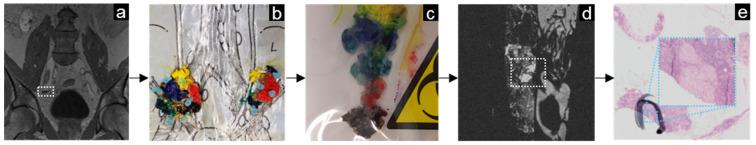
Example of the workflow in prostate cancer. In vivo MRI with a suspicious lymph node in the right obturator fossa ((**a**) white dashed lined box). Each nodal station was colored and pinned to an anatomical reference drawing to preserve orientation (**b**). The surgical specimen was vacuum-sealed to fixate the specimen for the ex vivo MRI (**c**). Due to a short USPIO-MRI interval of 1 day, USPIO particles were still present in the resection specimen. Since there was no loss of signal intensity in this lymph node, it was regarded as suspicious ((**d**) white dashed lined box). Pathology assessment with hematoxylin and eosin staining showed that this lymph node was metastatic (**e**).

**Figure 3:**
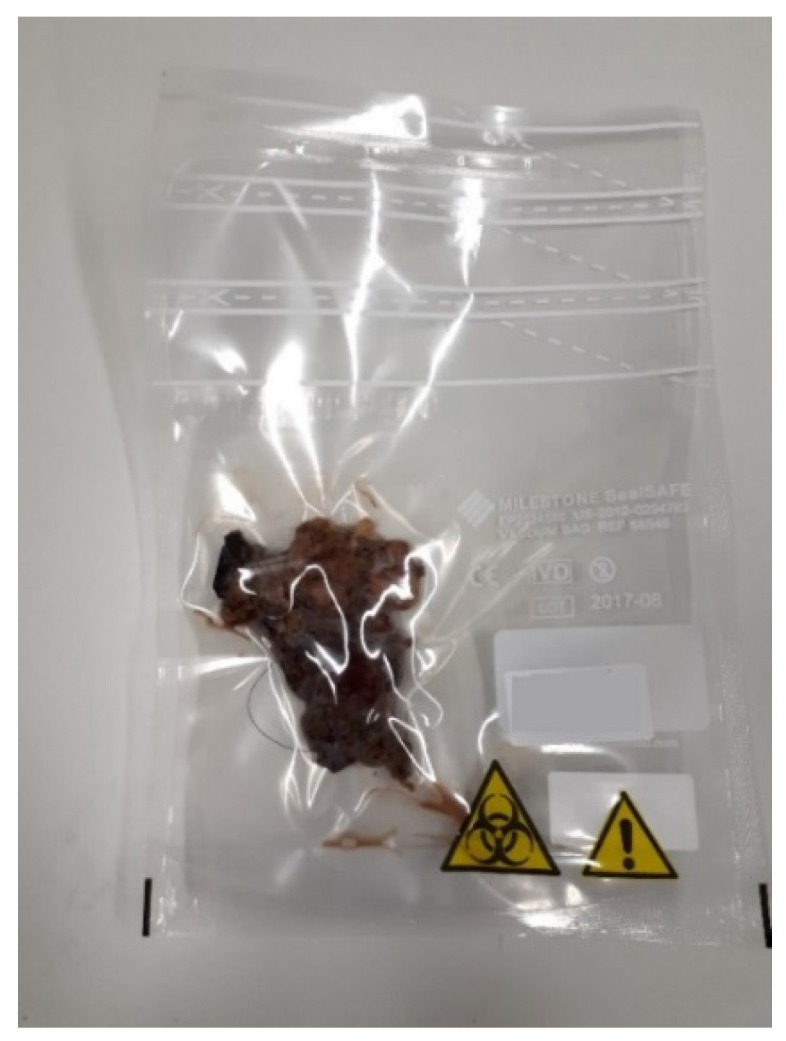
Example of a cervical lymphadenectomy specimen vacuum-sealed with the TissueSAFE plus device (Milestone, Sorisole, Italy).

A bilateral extended pelvic lymph node dissection was performed in the patient with prostate cancer for diagnostic purposes before treatment with radiotherapy. Each anatomical nodal station was separately dissected and pinned onto the corresponding region of a schematic anatomical plate (Figure 2). To preserve anatomical orientation, each nodal station was colored differently (Figure 2), and left- and right specimens were separately vacuum sealed using the TissueSAFE plus device (Milestone, Sorisole, Italy, Figure 3). Vacuum sealing the specimens offers several advantages: (1) the separate specimens of each nodal station are scanned in their original confirmation; (2) scanning the separate specimens together ensures that enough MR signal is generated; and (3) their orientation is preserved, and hence routine clinical practice is not disturbed. The use of a vacuum seal bag did not disturb ex vivo MR image quality. An ex vivo MRI of the vacuumed specimens was performed. The specimens were subsequently processed routinely at the dissection room; ex vivo MR images were not present during this process. The results of both the in vivo and ex vivo MRI were correlated to the final histopathological results on a basis-to-basis premise. In case ex vivo MRI yielded a higher number of lymph nodes than histopathological analysis, additional dissection of the specimen was performed to determine if the number of harvested evaluated lymph nodes can be increased.

### 3.2. Rectal Cancer

**Figure 4:**
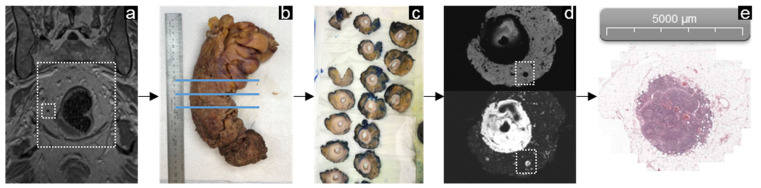
Example of the workflow in rectal cancer. A coronal view of the in vivo MRI showing the rectum within the white dashed lined box with a non-suspicious lymph node located right pararectally close to the rectal wall (**a**). After TME surgery, the specimen was fixated in formalin (**b**), of which an ex vivo MRI scan was performed. After the ex vivo scan, the specimen was sliced in 5 mm thick lamina slices (**c**), which can be compared to the transverse images of the ex vivo MRI (**d**). The lymph node was detected on ex vivo MRI as well as on pathology. Since the USPIO surgery interval was 9 days, and USPIO was no longer present in the resection specimen, and there were no changes in signal intensity. For this reason, the lymph node maintained a signal on the water-selective ex vivo MR image (**d**). Histopathological assessment with hematoxylin and eosin staining revealed a non-metastatic lymph node (**e**).

In the rectal cancer patient, surgical resection was performed according to the principle of a total mesorectal excision (TME) [21]. Lymph nodes that are likely to be involved in rectal cancer are located in the mesorectum, which is the fatty tissue surrounding the rectum bordered on the outside by the mesorectal fascia. This fascia forms an important anatomical barrier for tumor spread and is the intended surgical boundary of the TME procedure. Matching the ex vivo specimen to the in vivo appearance of the mesorectum after a well-performed surgical procedure is therefore relatively easy. Pathological assessment of the resected specimen is performed according to the method described by Quirke et al. [22]. The specimen was evaluated from distal to proximal by slicing serial transverse tissue lamina with a 5 mm interval. Each slice was examined for the presence of lymph nodes guided by the ex vivo MR images present at the dissection room. All nodes on in vivo MRI, ex vivo MRI, and histopathology were annotated, and matching was performed manually on the basis of location, size, and shape (Figure 4).

### 3.3. Periampullary Cancer

**Figure 5:**
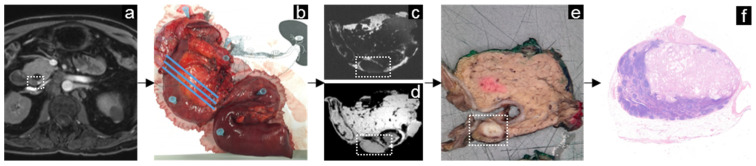
Example of the workflow in periampullary cancer. A transversal view of the T1 VIBE Dixon in vivo MRI showing the pancreas with a suspicious lymph node located dorsally from the pancreatic head in the lined box (**a**). After pancreaticoduodenctomy, the fresh resection specimen was pinned on an anatomical drawing (**b**), and an ex vivo MRI scan was performed, on which the same lymph node could be identified on the lipid-selective image (**c**) and the water-selective image (**d**). The USPIO-surgery interval was 8 days; thus, USPIO was no longer present in the resection specimen and therefore did not elicit any signal intensity changes. After the ex vivo MRI, the specimen was fixated and sliced into 5 mm thick slices (**e**). Histopathologic examination with hematoxylin and eosin staining revealed a metastasis within the lymph node (**f**).

In the patient with periampullary cancer, a pancreaticoduodenectomy with lymphadenectomy was performed. Most lymph nodes were resected en-bloc with the tumor; additionally, several lymph nodes were resected separately. After resection, the surgeon pinned the specimen onto an anatomical drawing displaying the pancreatic region, including its lymph node stations, of which a digital photograph was taken (Figure 5). At the Department of Pathology, the resection planes were inked, the primary tumor was incised, and the entire specimen was fixated with formalin [23]. The fixated specimen was processed routinely according to the axial slicing technique (Figure 5) [23]. Nodal correlation between in vivo MRI, ex vivo MRI, and histopathology was performed on a nodal station basis and, where possible, on a node-to-node basis.

### 3.4. Esophageal Cancer

**Figure 6:**
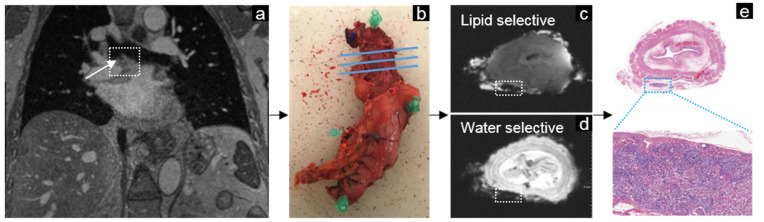
Example of the workflow in esophageal cancer. Lymph nodes were identified on the in vivo MRI after neoadjuvant chemoradiotherapy (**a**). Nodes could be matched using corresponding anatomical landmarks to the surgical specimen (**b**) and ex vivo MRI (**c**,**d**). The lipid-selective (**c**) and water-selective (**d**) ex vivo MRI guided the pathologist towards the lymph nodes for histopathological analysis of each node with hematoxylin and eosin staining (**e**). The white dashed lined box on the ex vivo MRI (**c**,**d**) and pathology (**e**) contains a healthy lymph node with USPIO contrast (the USPIO surgery interval was 1 day). Therefore, the lymph node lost its signal intensity on the water-selective image.

For the esophageal cancer patient, esophagectomy with two-field lymphadenectomy (thoracic and abdominal lymph node stations) was performed after neoadjuvant chemoradiotherapy. During surgery, the esophagus including locoregional lymph nodes was dissected en-bloc. The surgeon labeled all the lymph node stations within the surgical specimen with colored plastic beads, according to the TIGER study protocol [24]. Differences in the configuration of the ex vivo specimen as compared to its original in situ anatomical location made it difficult to correlate the lymph node stations on in vivo MRI and histopathology using anatomical landmarks. However, using the labeled location of the lymph node stations, lymph nodes on in vivo MRI were matched to ex vivo MRI and histopathology on a nodal, or at least a nodal station, basis (Figure 6).

### 3.5. Head-and-Neck Cancer

**Figure 7:**
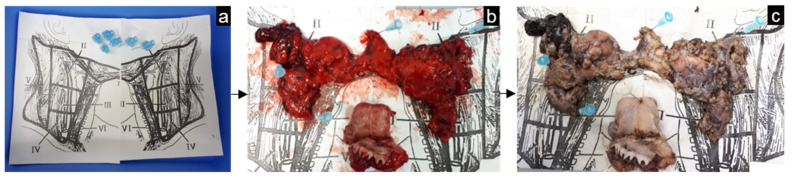
Example of tissue fixation in head-and-neck cancer. A grid containing a reference drawing of the neck including the levels (**a**) was present at the operating room. After resection, the fresh specimen was pinned to this grid (**b**) and was subsequently fixated with formalin at the Department of Pathology (**c**).

**Figure 8:**
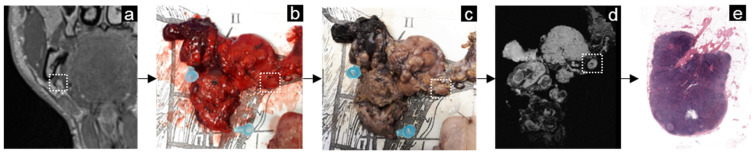
Example of the workflow in head-and-neck cancer. A lymph node was located on the in vivo MR image ((**a**) white dashed lined box). After lymphadenectomy, the fresh specimen was pinned to a grid (**b**) and fixated with formalin (**c**). Of the fixated specimen, an ex vivo MRI was performed. The lymph node of interest identified on the in vivo MR image was correlated to the ex vivo MR image (**d**). Since the USPIO surgery interval was 5 days, and USPIO was no longer present in the resection specimen, there were no changes in signal intensity. For this reason, the lymph node maintained a signal on the water-selective ex vivo MR image (**d**). The pathologist was guided by the ex vivo MRI to dissect this node and enclose it separately. Histopathologic examination with hematoxylin and eosin staining revealed a healthy lymph node (**e**).

In the head-and-neck cancer patient, the neck dissection specimen was taken out en-bloc. The primary tumor was resected separately. After resection, the specimen was pinned to a grid with a reference drawing of the neck, including its anatomical nodal levels, and fixated with formalin (Figure 7 and Figure 8). Next, the different neck levels were separated. To maintain orientation, pieces were vacuum-sealed in their original configuration with a TissueSAFE plus system (Figure 3) and scanned ex vivo in the 7T preclinical MR system. The cuts, or the boundaries between the neck levels, were visible on ex vivo MRI. All suspicious nodes were individually matched on in vivo MRI, ex vivo MRI, and histopathology. These nodes were cut into multiple sections and stained with immunohistochemistry in addition to standard hematoxylin and eosin staining. All non-suspicious nodes were routinely dissected and processed per neck level. Therefore, this workflow enabled correlation on a nodal basis for suspicious nodes and correlation on a nodal station basis for non-suspicious nodes.

## 4. Discussion

We developed a method that enabled the correlation of in vivo-detected lymph nodes from different primary solid malignancies with histopathology. The workflow was technically feasible in prostate, rectal, periampullary, esophageal, and head-and-neck cancer patients. The intermediate step of the 7T ex vivo MRI scan of resected specimens allowed in principle correlation on a nodal basis, but at least on a nodal station basis between the in vivo MRI images and histopathology. For each tumor location, different approaches were required to maintain an anatomical orientation of the resected specimen and correctly match the results to the pathological evaluation. In esophageal, periampullary, and rectal cancer, lymph nodes were dissected en-bloc with the primary tumor. Therefore, lymph nodes could be matched using corresponding anatomical landmarks to the ex vivo MRI. In head-and-neck and prostate cancer, lymphadenectomy and primary tumor dissection were performed separately. Hence, the lymphadenectomy specimens were vacuum-sealed in plastic bags in order to maintain orientation and easy positioning of the specimen within the scanner.

### 4.1. Comparison with the Literature

Few attempts have been made to match individual lymph nodes visible on MRI with their precise histologic matching after lymphadenectomy. For rectal cancer, Park et al. proposed a method where ex vivo ultrasound was used to match in vivo MRI with histopathology [16]. The use of identical scanning parameters for both in vivo MRI and ex vivo ultrasound enabled successful matching on a nodal basis in 91% of the lymph nodes. However, only lymph nodes >3 mm were depicted with MRI and matched with histopathology. A recent study applied ultrahigh field ex vivo MRI of TME specimens in 22 rectal cancer patients and demonstrated that the mean size of the lymph nodes found on ex vivo MRI was below 3 mm [20]. Luciani et al. [17] studied 1.5T ex vivo MRI for precise node-to-node correlation in female breast cancer patients, but 20% of nodes found with pathology were not depicted with ex vivo MRI. A likely explanation is that the used magnetic field strength was only 1.5T, MR sequences were 2-dimensional, and slice thickness was 2-3 mm, easily missing the smaller sized nodes. By comparison in the workflow presented here, ex vivo MRI was performed with 3D sequences and a partition/slice thickness of 0.29 mm at a field strength of 7T, most likely improving nodal yield on MRI. Korteweg et al. examined healthy axillary lymphoid tissue of two deceased females on a clinical 7T ex vivo MRI system and detected lymph nodes <1 mm, corroborating our results [4]. Both studies [4,17] proposed a framework for exact matching of radio-pathological findings by pinning the dissected specimen to an MR compatible grid containing an explanatory abscissa and ordinate [17] or vertical and horizontal reference lines [4]. For each lymph node, the in-plane position was assessed and correlated to the MR images. Matching was successful in 80% [17] and 88% [4] of the lymph nodes, although this percentage strongly depends on the total amount of identified lymph nodes.

### 4.2. Strengths and Limitations

We developed a workflow to match individual lymph nodes detected on in vivo MRI to the resection specimen by incorporating 7T ex vivo MRI into the protocol. This method can be used in studies validating MRI, such as USPIO-enhanced MRI, for detection of lymph node metastases on a nodal basis with histopathology as the gold standard. The method was illustrated in prostate, rectal, periampullary, esophageal, and head-and-neck cancer. The results demonstrate that various approaches enabled maintenance of orientation in both large, small, and anatomically complex resection specimens. Therefore, the presented workflow can easily be adapted to other organs. High-resolution ex vivo MRI provides direct information on the location of lymph nodes in resection specimens and is of immediate help to harvest nodes in general, or suspicious nodes in particular. In cases of a short time interval between USPIO-enhanced MRI and surgery, maintenance of signal intensity on the ex vivo MR images represents the absence of USPIO deposits and thus a lymph node suspicious for harboring a metastasis. This functional information can be of added value for node-to-node correlation. Radiologic differentiation between a lymph node and a blood vessel was formerly potentially difficult. However, because of the use of 3D high-resolution MR sequences enabling visualization of nodal structures in the transverse, sagittal, and coronal planes, this is no longer an issue.

Some potential limitations should also be mentioned. Node-to-node correlation for all lymph nodes is challenging. Differences in spatial resolution between in vivo and ex vivo exams can lead to differences in detection of lymph nodes, particularly small ones. Additionally, there could be a difference between the resected volume and a volume defined as resected on in vivo images, which can also lead to differences in the number of evaluated lymph nodes. Moreover, the fixation process needed for the pathology processing causes volume change of the specimen, which leads to a change of configuration of the specimen and landmarks. Neck dissection and pelvic lymph node dissection specimens, for example, are relatively small but harbor many lymph nodes and generally contain only a few anatomical landmarks. Therefore, it is of paramount importance that the anatomical orientation of the specimen is well documented during dissection. In this way, a reliable node-to-node correlation can be achieved, or in the case of clusters of small lymph nodes, a per nodal station correlation can be performed. Nevertheless, node-to-node correlation for all lymph nodes detected on in vivo MRI remains challenging. For rectal cancer, only 55 lymph nodes of the 216 removed during pathological examination could be correlated on a node-to-node basis [16]. Hence, the approach described for esophageal cancer and head-and-neck cancer, in which only the suspicious lymph nodes are correlated on a nodal basis and the remaining non-suspicious lymph nodes are correlated on a nodal station basis, might be more achievable and therefore more reliable. Moreover, clinical decisions are often based on a nodal station basis rather than on a nodal station. Therefore, in the clinical setting, correlation on a nodal level is less relevant. In a research setting, an extensive validation of all (smallest) lymph nodes will remain very time-consuming and challenging: at the start of pathological examination of resected tissue, the first nodes can be matched and excised with high precision, but further handling of the tissue towards multiple other nodes will inevitably make matching between ex vivo MRI and individual nodes in the remaining tissue more difficult. In addition, the ex vivo MRI requires a considerable investment of time which is not always desirable in clinical practice. Likewise, not every institution has a 7T preclinical MR system available for ex vivo measurements. Therefore, the method we developed is particularly suitable for studies validating MRI techniques for nodal assessment.

### 4.3. Clinical Implications 

In various types of cancer, detection of small lymph node metastases is urgently needed. Both over- and undertreatment in oncological therapeutic regimens result in unnecessary morbidity and mortality. Treatment needs to be tailored on a patient basis to overcome this problem. Thus, knowledge regarding merely the presence (N+) or absence (N0) of nodal metastases is not sufficient anymore. Clinicians need to be informed about the number, size, and exact location of metastatic deposits, which is a prerequisite to target therapies. Individual treatment strategies consist of local tumor excision or ablation without lymphadenectomy in N0 patients, reducing unnecessary morbidity in this group of patients. In N+ cancer patients, the exact N-staging can lead to additional treatment options for local lymph node metastases such as surgical resection, ablation, or image-guided radiotherapy. Currently, a variety of promising non-invasive diagnostic imaging tests are developed to detect lymph node metastases, e.g., hybrid PET-MRI, USPIO-enhanced MRI, and targeted fluorescence imaging [5,25,26]. Using our described workflow with 7T ex vivo MRI as an intermediate step to guide histopathological work-up, new techniques can be validated on a node-to-node basis with histopathology. The detailed 7T ex vivo MRI of the resection specimen aids in finding the smallest lymph nodes that otherwise might be missed with routine pathological evaluation [20], supporting the validation of in vivo imaging techniques. 

## 5. Conclusions

A technically feasible method was developed to correlate lymph nodes identified on USPIO-enhanced MRI for the detection of lymph node metastases with histopathology of the same nodes as gold standard. This workflow was suitable in various anatomical regions. Ex vivo MRI-guided pathological dissection enabled correlation of visible lymph nodes on in vivo MR images of various body regions with histopathology of resected lymph nodes on a nodal, or at least a nodal, station basis. This approach allows for the validation of in vivo USPIO-enhanced MRI for evaluation of locoregional lymph nodes, which is essential for a subsequent diagnostic accuracy assessment.

## Figures and Tables

**Table 1:** Patient characteristics.

Patient	Sex	Age	Cancer Type	TNM-Stage	Therapy	USPIO Infusion-Surgery Interval	Study Registration Number
1.	Male	67	Prostate	cT3N1M0	Pelvic lymphadenectomy followed by post-operative radiotherapy	1 day	None
2.	Male	76	Rectum	cT2N0	Total mesorectal excision	9 days	NCT02751606
3.	Male	58	Periampullary	p T2N2M1 *	Pancreaticoduodenectomy with lymphadenectomy followed by postoperative chemotherapy	8 days	NCT04311047
4.	Female	65	Esophagus	cT2-3N1	Neoadjuvant chemoradiotherapy followed byesophagectomy	1 day	NTR6072
5.	Male	51	Head-and-neck	cT3N0M0	Primary tumor resection and cervical lymphadenectomy followed by postoperative radiotherapy	5 days	NCT03817307

Abbreviations: c = clinical, p = pathological, TNM = tumor node metastasis, USPIO = ultrasmall superparamagnetic iron oxide. * This patient had metastatic distal lymph nodes, which are considered as M1 according to the TNM 8th edition.

**Table 2:** Overview of parameters regarding the in vivo T1-weighted MR sequences.

	Head-and-Neck Cancer	Esophageal Cancer	Periampullary Cancer	Rectal Cancer	Prostate Cancer
Sequence	VIBE Dixon	VIBE Dixon	VIBE Dixon	VIBE Dixon	VIBE
Voxel size (mm^3^)	0.8 × 0.8 × 0.8	1.4 × 1.4 × 1.4	1.4 × 1.4 × 1.7	0.9 × 0.9 × 0.9	0.73 × 0.89 × 0.91
FOV (mm^3^)	260 × 260 × 154	450 × 394 × 168	394 × 450 × 177	350 × 350 × 173	328 × 328 × 175
Acquisition mode	3D	Breath-hold 3D	Breath-hold 3D	3D	3D
TE (ms)	2.57, 3.8	1.23, 2.46	1.23, 2.46	1.2, 2.5	2.5
TR (ms)	6.02	4.21	3.86	5.8	6.5
Bandwidth (Hz)	300	20	20	305	510
Acquisition time (min)	4:53	18 s for each breath-hold	12 s for each breath-hold	4:56	9:13
Flip angle (°)	10	9	9	10	10

Abbreviations: 3D = 3-dimensional, FOV = field of view, Hz = Hertz, min = minutes, ms = milliseconds, TE = echo time, TR = repetition time, VIBE = volumetric interpolated breath-hold examination.

**Table 3:** Overview of parameters regarding the in vivo T2*-weighted iron-sensitive MR sequences.

	Head-and-Neck Cancer	Esophageal Cancer	Periampullary Cancer	Rectal Cancer	Prostate Cancer
Sequence	mGRE	mGRE	mGRE	mGRE	MEDIC
Voxel size (mm^3^)	0.8 × 0.8 × 0.7	1.5 × 1.5 × 1.5	1.3 × 1.3 × 1.3	0.85 × 0.85 × 0.85	0.73 × 0.73 × 0.73
FOV (mm^3^)	260 × 260 × 154	336 × 279 × 30	291 × 291 × 211	328 × 328 × 190	328 × 328 × 164
Acquisition mode	3D	Breath-hold 3D	Breath-hold 3D	3D	3D
Acquired TEs (ms)	2.5–27.1	2.7–16.7	2.7–16.7	3.6–17.2	12
Reconstructed TEs (ms)	12	12	12	12	n.a.
TR (ms)	31	20	20	590	21
Bandwidth (Hz)	360	380	380	3 × 1	172
Acquisition time (min)	11:08	19 s each Breath-hold	20 s eachBreath-hold	11:28	12:27
Flip angle (°)	10	10	10	10	10

Abbreviations: 3D = 3-dimensional, FOV = field of view, Hz = Hertz, MEDIC = multiple echo data image combination, mGRE = multi-gradient echo sequence, min = minutes, ms = milliseconds, n.a. = not applicable, TE = echo time, TR = repetition time.

**Table 4:** Overview of ex vivo MR parameters (7 Tesla, Bruker ClinScan).

Ex Vivo MRI	3D Lipid Excitation	3D Water Excitation
Acquisition time (min)	13	20
TR (ms)	15	30
TE (ms)	3.0	6.2
Resolution (mm^3^)	0.29 × 0.29 × 0.29	0.29 × 0.29 × 0.29
Flip angle (°)	10	14

Abbreviations: 3D = 3-dimensional, min = minutes, MRI = magnetic resonance imaging, ms = milliseconds, TE = echo time, TR = repetition time.

## Data Availability

The data are not publicly available due to them containing information that could compromise research participant privacy.
